# Long-term morbidity and mortality in patients diagnosed with an insulinoma

**DOI:** 10.1530/EJE-21-0230

**Published:** 2021-09-01

**Authors:** Elina Peltola, Päivi Hannula, Heini Huhtala, Saara Metso, Juhani Sand, Johanna Laukkarinen, Mirja Tiikkainen, Jukka Sirén, Minna Soinio, Pirjo Nuutila, Leena Moilanen, David E Laaksonen, Tapani Ebeling, Johanna Arola, Camilla Schalin-Jäntti, Pia Jaatinen

**Affiliations:** 1 Faculty of Medicine and Health Technology, Tampere University, Tampere, Finland; 2 Division of Internal Medicine, Seinäjoki Central Hospital, Seinäjoki, Finland; 3 Endocrinology, Department of Internal Medicine, Tampere University Hospital, Tampere, Finland; 4 Faculty of Social Sciences, Tampere University, Tampere, Finland; 5 Department of Gastroenterology and Alimentary Tract Surgery, Tampere University Hospital, Tampere, Finland; 6 Endocrinology, Abdominal Center; 7 Surgery, Abdominal Center, Helsinki University Hospital, Helsinki, Finland; 8 Surgery, Abdominal Center, University of Helsinki, Helsinki, Finland; 9 Department of Endocrinology, Division of Medicine, Turku University Hospital, Turku, Finland; 10 Department of Endocrinology, Division of Medicine, Turku University Hospital, Turku, Finland; 11 Turku PET Centre, University of Turku, Turku, Finland; 12 Department of Medicine, Kuopio University Hospital, Kuopio, Finland; 13 Faculty of Medicine, University of Oulu, Oulu, Finland; 14 Endocrinology, Department of Medicine, Oulu University Hospital, Oulu, Finland; 15 Pathology, HUSLAB, Helsinki University Hospital, Helsinki, Finland; 16 Pathology, University of Helsinki, Helsinki, Finland; 17 Endocrinology, Abdominal Center, University of Helsinki, Helsinki, Finland

## Abstract

**Objective:**

Insulinomas are rare functional pancreatic neuroendocrine tumours. As previous data on the long-term prognosis of insulinoma patients are scarce, we studied the morbidity and mortality in the Finnish insulinoma cohort.

**Design:**

Retrospective cohort study.

**Methods:**

Incidence of endocrine, cardiovascular, gastrointestinal and psychiatric disorders, and cancers was compared in all the patients diagnosed with an insulinoma in Finland during 1980–2010 (*n* = 79, including two patients with multiple endocrine neoplasia type 1 syndrome), vs 316 matched controls, using the Mantel–Haenszel method. Overall survival was analysed with Kaplan–Meier and Cox regression analyses.

**Results:**

The median length of follow-up was 10.7 years for the patients and 12.2 years for the controls. The long-term incidence of atrial fibrillation (rate ratio (RR): 2.07 (95% CI: 1.02–4.22)), intestinal obstruction (18.65 (2.09–166.86)), and possibly breast (4.46 (1.29–15.39) and kidney cancers (RR not applicable) was increased among insulinoma patients vs controls, *P*  < 0.05 for all comparisons. Endocrine disorders and pancreatic diseases were more frequent in the patients during the first year after insulinoma diagnosis, but not later on. The survival of patients with a non-metastatic insulinoma (*n* = 70) was similar to that of controls, but for patients with distant metastases (*n* = 9), the survival was significantly impaired (median 3.4 years).

**Conclusions:**

The long-term prognosis of patients with a non-metastatic insulinoma is similar to the general population, except for an increased incidence of atrial fibrillation, intestinal obstruction, and possibly breast and kidney cancers. These results need to be confirmed in future studies. Metastatic insulinomas entail a markedly decreased survival.

## Introduction

Insulinomas are rare insulin-secreting functional pancreatic neuroendocrine tumours, with an estimated incidence of 1–4 per million per year (1, 2, 3, 4). They usually do not show metastatic behaviour and are considered cured after complete surgical removal of the tumour (3, 4, 5). On the other hand, disease recurrence occurs in 7% of the surgically treated patients (4), and in patients with distant metastases, the median survival has been reported to be less than 2 years (2).

Despite the improved diagnostic options, the diagnostic delay of insulinomas has remained long, presumably due to the rarity of the disease and the nonspecific clinical picture, as we have shown in our previous study on all adult patients diagnosed with an insulinoma in Finland during 1980–2010 (6). Because previous data on the long-term prognosis of insulinoma patients are scarce, we wanted to study the long-term morbidity and mortality in the Finnish insulinoma cohort (6).

## Subjects and methods

The Finnish insulinoma register consists of all adult patients (≥18 years of age) diagnosed with an insulinoma in Finland during 1980–2010 (*n* = 79) (6). For each patient, four controls were chosen from the Finnish Population Register Centre. The controls had to be equal by age (±6 months), gender, and the place of residence, and alive at the diagnosis of the corresponding patient. The dates of death or emigration were provided by the Finnish Population Register Centre. Personal identification numbers assigned to all Finnish residents were used to link the information from the separate registers described below. The register-based follow-up began on 1 January 1980 and lasted until 31 December 2015, unless death or emigration occurred first.

The morbidity of insulinoma patients vs controls was analysed before and after the diagnosis of insulinoma, focusing on five distinct disease groups: endocrine, gastrointestinal, cardiovascular, cancer, and psychiatric diseases, to evaluate the potential comorbidity and long-term effects of insulinoma on the development of these diseases. Cancer morbidity was evaluated based on the cancer diagnoses registered at the Finnish Cancer Registry, where Finnish health care organizations statutorily provide information on all new cancer cases. Insulinoma-related notifications were excluded from the analyses.

Morbidity due to endocrine, cardiovascular, gastrointestinal, and psychiatric disorders was evaluated on the basis of the diagnoses registered at the National Hospital Discharge Register, the Care Register for Health Care. This register, maintained by the Finnish Institute of Health and Welfare, collects the statutory data on all Finnish residents discharged from inpatient care in any Finnish hospital since 1969 and on outpatient visits in specialized health care since 1998. The diagnoses are coded according to the Finnish version of the 10th revision of the International Classification of Diseases (ICD-10) since 1996, ICD-9 during 1987–1995, and ICD-8 during 1980–1986. These classifications were reviewed, and the diagnoses of interest were grouped into corresponding disease categories and subcategories (Supplementary Table 1, see section on supplementary materials given at the end of this article). Both the primary and the secondary diagnoses were included in the analyses, and the diagnosis codes for hyperinsulinism and hypoglycaemia were excluded from the analysis of endocrine disorders.

For the mortality analyses, the causes of death were obtained from Statistics Finland, which collects the dates and causes of death of all Finnish citizens deceased since 1971. The causes of death are classified according to the ICD, as well as with a national time series classification, including 54 categories. In the analyses, we used the underlying cause of death, defined as the disease that has initiated the series of illnesses directly leading to death.

This study was conducted in accordance with the Declaration of Helsinki. The Regional Ethics Committee of the Tampere University Hospital catchment area reviewed and approved the study protocol. Informed consent was waived because of the retrospective, register-based nature of the study, and the fact that many of the study subjects died before data collection for the study. The Finnish Institute for Health and Welfare, the University Hospitals of Tampere, Helsinki, Kuopio, Oulu and Turku, Statistics Finland, and the Finnish Population Register Centre yielded permission for the use of data from their registers. Research data are not shared for ethical reasons, to protect the anonymity of patients with a rare disease.

### Statistical analysis

The analyses were conducted with the IBM SPSS Statistics for Windows, Versions 25.0 and 27.0 (IBM Corp.), the STATA Statistical Software, Release 13 (StataCorp LP), and the OpenEPI Collection of Epidemiologic Calculators, Version 3.01. The data are presented as mean (s.d.) for normally distributed variables, median (minimum–maximum) for other numerical variables, and number (%) for categorical variables.

In the morbidity analyses, the prevalence of diseases diagnosed before the diagnosis of insulinoma was first compared between the patients and the controls with the Fisher’s exact test and conditional logistic regression. Then, the incidence rates of these disease groups after the diagnosis of insulinoma were compared by analysing the incidence rate ratios (RR) and 95% CIs, using the Mantel–Haenszel method. Because only the first notification of each disease category per person was included in the analyses, the patients with a disease registered before the diagnosis of insulinoma were excluded from the incidence calculations of that disease category, together with their controls. The controls with a given disease diagnosed before the index date were excluded from the analyses individually. For diseases with a statistically significant difference in the patients vs controls, a sensitivity analysis was performed, excluding the MEN1 patients and their controls, as well as the persons with each disease diagnosed within the first year after insulinoma diagnosis, to eliminate detection bias. The Bonferroni correction was applied to define the level of significance for multiple comparisons.

The overall survival of the patients vs controls was compared using Kaplan–Meier analysis with the log-rank test. Insulinomas were retrospectively classified according to their highest diameter (≥ vs <2 cm) and staged according to the most recent TNM classification system (7). Cox regression analyses were used to calculate the hazard ratios (HR), to identify factors associated with mortality among the patients. The distribution of the causes of death was compared with the Fisher’s exact test. For the patients who underwent curative-intent surgery, disease-free survival was calculated from the date of primary surgery to the date of disease progression or relapse. In the survival analyses, a two-sided *P* value below 0.05 was considered statistically significant.

## Results

Follow-up data of all the 79 patients and their 316 controls were included in the study. The mean age at the insulinoma diagnosis was 51.7 (15.6) years in the patients and 51.7 (15.5) years in the controls. The median duration of symptoms before the diagnosis was 13.0 (0.1–243.5) months. The median duration of the register-based follow-up between 1 January 1980, and the date of diagnosis of insulinoma was 22.7 (0.5–30.8) years for both the patients and the controls, and the median duration of follow-up after the diagnosis of insulinoma was 10.7 (0.2–32.6) years for the patients and 12.2 (1.2–35.5) years for the controls. A metastatic insulinoma was detected in nine (11%) patients. Multiple endocrine neoplasia type 1 (MEN1) syndrome was diagnosed in two patients, both associated with a non-metastatic, solitary insulinoma.

### Long-term morbidity

**Figure 1:**
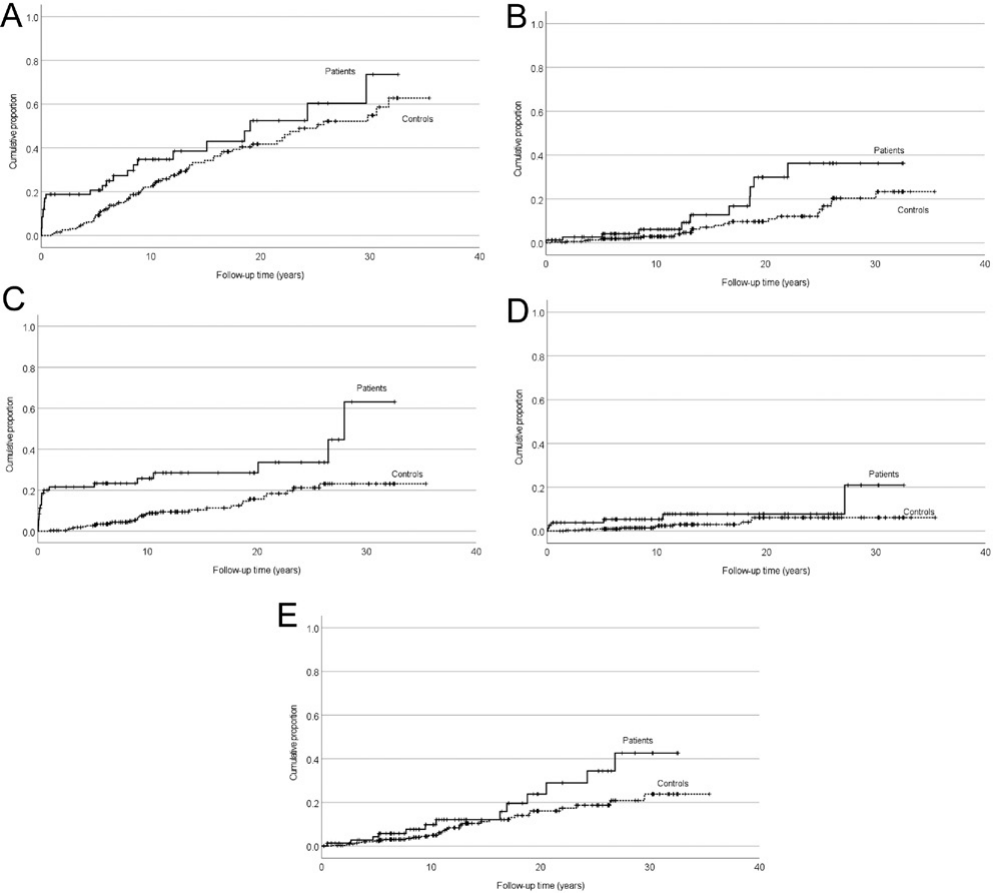
(A, B, C, D and E) Cumulative incidence of cardiovascular, endocrine, and cancer diseases in the patients (solid line) diagnosed with an insulinoma Finland during 1980–2010, compared with controls (dashed line) matched for age, gender, and the place of residence (log-rank test). (A) Cardiovascular diseases (*P*  = 0.048), (B) atrial fibrillation (*P*  = 0.024), (C) endocrine disorders (*P*  < 0.001), (D) thyroid disorders (*P* = 0.047), (E) cancers (*P*  = 0.061).

Before the diagnosis of insulinoma, there was no statistically significant difference in morbidity between the patients and the controls (Supplementary Table 2). After the diagnosis of insulinoma, the overall incidence of any cardiovascular disease and the incidence of atrial fibrillation were increased in the patients vs controls, although the increase was not significant after the Bonferroni correction (Fig. 1A and Table 1). In the Kaplan–Meier analysis, the difference in the cumulative incidence of atrial fibrillation in the patients vs controls increased gradually after the diagnosis of insulinoma (Fig. 1B). A sensitivity analysis excluding the first year after insulinoma diagnosis showed a trend towards an increased incidence of atrial fibrillation in the insulinoma patients, but this difference was not statistically significant (Table 2).

**Table 1:** Incidence of endocrine, cardiovascular, gastrointestinal, and psychiatric disorders in the 79 patients diagnosed with an insulinoma in Finland during 1980–2010, and in 316 matched controls, after the diagnosis of insulinoma.

Disease	Patients			Controls			Patients vs controls		*P* value
	*n*/*n* at risk	Incidence rate/10 000/year	95% CI	*n*/*n* at risk	Incidence rate/10 000/year	95% CI	Rate ratio	95% CI	
Endocrine disorders^a^	21/72	295.37	192.58–453.01	32/273	84.58	59.81–119.60	3.49	2.01–6.06	**<0.001**
Diabetes	8/75	89.05	44.53–178.07	22/294	53.36	35.14–81.04	1.67	0.74–3.75	0.210
Thyroid disorders	6/79	61.72	27.73–137.39	10/311	22.35	12.02–41.55	2.76	1.00–7.60	**0.040**
Parathyroid disorders	2/79	20.16	5.04–80.61	0/315	0.00	0.00–7.97	NA		
Other endocrine disorders^b^	11/76	130.00	70.65–230.00	1/301	2.27	0.32–16.14	56.12	7.25–434.67	**<0.001**
Cardiovascular diseases	24/59	423.26	283.70–631.47	70/196	265.79	210.28–335.95	1.59	1.00–2.53	**0.047**
Cerebrovascular diseases	7/78	74.27	35.41–155.79	29/305	67.92	47.20–97.73	1.09	0.48–2.50	0.832
Hypertension	15/70	189.95	114.51–315.07	47/265	123.68	92.93–164.61	1.54	0.86–2.75	0.145
Arrhythmias and conduction disorders	15/77	166.75	100.53–276.59	40/299	93.64	68.69–127.65	1.78	0.98–3.22	0.053
Atrial fibrillation and flutter	11/78	117.74	65.20–212.60	25/307	56.75	38.35–83.99	2.07	1.02–4.22	**0.039**
Coronary artery disease	11/77	119.17	66.00–215.19	36/297	87.03	62.77–120.65	1.37	0.70–2.69	0.360
Diseases of the arteries and veins	7/73	78.92	37.62–165.54	30/259	81.56	57.02–116.64	0.97	0.43–2.20	0.938
Valvular diseases and cardiomyopathies	4/76	42.12	15.81–112.23	12/302	27.24	15.47–47.96	1.55	0.50–4.80	0.447
Heart failure	4/77	40.03	15.02–106.66	19/304	42.87	27.34–67.21	0.93	0.32–2.75	0.901
Diseases of the pulmonary circulation	2/79	19.72	4.93–78.84	4/316	8.77	3.29–23.36	2.25	0.41–12.28	0.336
Gastrointestinal diseases	27/67	463.70	318.00–676.16	55/233	175.53	134.76–228.63	2.64	1.67–4.19	**<0.001**
Diseases of the oesophagus, stomach, and duodenum	6/76	63.83	28.68–142.07	22/298	51.70	34.04–78.52	1.24	0.50–3.05	0.647
Abdominal hernias^c^	8/73	91.32	45.67–182.61	19/282	45.96	29.31–72.05	1.99	0.87–4.54	0.097
Chronic inflammatory bowel diseases	0/79	0.00	0.00–35.85	3/315	6.58	2.12–20.39	0.00		0.412
Diseases of the appendix	2/78	20.46	5.12–81.79	8/304	18.32	9.16–36.63	1.12	0.24–5.26	0.889
Other bowel diseases	14/77	156.41	92.63–264.09	30/296	71.68	50.11–102.51	2.18	1.16–4.12	**0.013**
Diseases of the liver, biliary tract, and gallbladder	6/77	65.02	29.21–144.72	20/292	48.18	31.08–74.68	1.35	0.54–3.36	0.518
Diseases of the pancreas^d^	8/78	85.62	42.82–171.20	3/311	6.66	2.15–20.64	12.86	3.41–48.49	**<0.001**
Mental and behavioural disorders	12/71	148.85	84.54–262.11	36/265	98.09	70.76–135.99	1.52	0.79–2.92	0.208
Dementia	4/79	39.64	14.88–105.61	14/312	30.86	18.28–52.11	1.28	0.42–3.90	0.658

Bold values indicate a statistically significant difference between the patients and the controls (*P*  < 0.05, Mantel–Haenszel method). When the Bonferroni correction for multiple comparisons is applied, a *P* value < 0.002 (<0.05/25) is considered statistically significant.

^a^Excluding hyperinsulinism and hypoglycaemia; ^b^Other endocrine disorders in the patients included a polyglandular endocrine disorder (*n* = 1) and other or unspecified endocrine disorders (*n* = 10); ^c^Abdominal hernias in the patients included seven ventral hernias and one inguinal hernia; ^d^Pancreatic diseases in the patients included acute pancreatitis (*n* = 6), pancreatic pseudocyst (*n* = 1), and other/undefined pancreatic disease (*n* = 1).

NA, not applicable.

**Table 2:** Sensitivity analysis of the incidence of endocrine, cardiovascular, gastrointestinal, and psychiatric disorders in the 77 patients diagnosed with a sporadic insulinoma in Finland during 1980–2010, and in 308 matched controls, after the diagnosis of insulinoma, excluding the first year after diagnosis.

Disease	Patients^a^			Controls			Patients vs controls		*P*-value
*n*/*n* at risk^b^	Incidence rate/10 000/year	95% CI	*n*/*n* at risk^b^	Incidence rate/10 000/year	95% CI	Rate ratio	95% CI
Endocrine disorders^c^	7/52	107.19	51.10–224.85	27/219	91.76	62.93–133.80	1.17	0.51–2.68	0.714
Thyroid disorders	3/69	34.88	11.25–108.13	9/296	22.87	11.90–43.96	1.53	0.41–5.63	0.524
Parathyroid disorders	1/72	11.07	1.56–78.59	0/308	0.00		NA		
Other endocrine disorders	1/61	12.56	1.77–89.13	1/262	2.74	0.39–19.43	4.59	0.29–73.34	0.236
Cardiovascular diseases	12/44	220.51	125.23–388.29	44/147	227.49	169.29–305.69	0.97	0.51–1.84	0.924
Atrial fibrillation and flutter	10/70	121.66	65.46–226.11	20/274	58.34	37.64–90.42	2.09	0.98–4.46	0.052
Gastrointestinal diseases	13/46	262.06	152.16–451.31	38/175	169.19	123.11–232.51	1.55	0.83–2.91	0.170
Other bowel diseases	11/68	138.54	76.72–250.16	26/279	70.39	47.93–103.38	1.97	0.97–3.98	0.055
Intestinal obstruction	4/72	46.07	17.29–120.00	1/307	2.43	0.34–17.25	18.96	2.12–169.64	**<0.001**
Diseases of the pancreas	1/66	11.99	1.69–85.09	3/263	9.04	2.91–28.02	1.33	0.14–12.75	0.806

Bold value indicates a statistically significant difference between the patients and the controls (*P*  < 0.05, Mantel–Haenszel method). When the Bonferroni correction for multiple comparisons is applied, a *P* value <0.005 (<0.05/10) is considered statistically significant.

^a^Two patients with MEN1 syndrome were excluded from the analyses, together with their corresponding controls; ^b^Patients with a disease diagnosed before or within 1 year after the diagnosis of insulinoma were excluded from the incidence calculations of that disease category, together with their corresponding controls. Controls with a given disease diagnosed before or within 1 year after the diagnosis of insulinoma of the corresponding patient, as well as patients and controls with an insufficient follow-up time (less than a year after the diagnosis of insulinoma) were excluded individually; ^c^Excluding hyperinsulinism and hypoglycaemia.

NA, not applicable.

Regarding endocrine morbidity (Fig. 1C), the overall incidence of endocrine and thyroid disorders was higher in the patients than in the controls (Fig. 1C and Table 1). The thyroid diagnoses included hypothyroidism in three (4%), hyperthyroidism in two (3%), and goitre in one (1%) of the patients, compared to six (2%), two (1%), and two (1%) of the controls, respectively. In the Kaplan–Meier analysis, the cumulative incidence of thyroid disorders in the patients vs controls started to increase right from the diagnosis of insulinoma (Fig. 1D). After excluding the first post-diagnostic year, no difference was found in the incidence of endocrine or thyroid disorders between the patients and the controls (Table 2). A parathyroid disorder (hyperparathyroidism) was diagnosed in only two (3%) patients, one of them having a confirmed MEN1 syndrome.

As for gastrointestinal diagnoses, the incidence of pancreatic diseases, and bowel diseases other than IBD, hernias, and appendiceal diseases, was increased among the insulinoma patients (Table 1). The increased incidence of pancreatic diseases was explained by acute pancreatitis, usually diagnosed during the first 2 months after the primary pancreatic surgery. In the subgroup analysis of bowel diseases, the only statistically significant difference was an increased incidence of intestinal obstruction, diagnosed in 5% of the surgically treated patients, a median of 5.9 (2.1–11.3) years after primary pancreatic surgery (RR: 18.7 (95% CI: 2.1–166.9), *P*  < 0.001).

The incidence of dementia or all mental and behavioural disorders did not significantly differ between the patients and the controls (Table 1). Regarding cancer morbidity, 14 cancers were diagnosed in the patients and 42 in the controls (Fig. 1E and Table 3), after the diagnosis of insulinoma. Of specific cancer types, the incidence of breast and kidney cancers was increased in insulinoma patients vs controls (Table 3). After the exclusion of the 2 MEN1 patients and their controls, however, no statistically significant increase was found in breast cancer incidence (RR 2.64 (0.63–11.04), *P*  = 0.167). The breast cancers occurred 4.7–24.3 years after the diagnosis of insulinoma. The three kidney cancers in the patients were diagnosed 2.7, 16.9, and 20.5 years after the diagnosis of a sporadic, non-metastatic insulinoma. Only one of the kidney cancers was detected before the end of insulinoma follow-up at the University Hospital.

**Table 3:** Cancer incidence in the 79 patients diagnosed with an insulinoma in Finland during 1980–2010, and in 316 matched controls, after the diagnosis of insulinoma.

Cancer type	Patients			Controls			Patients vs controls		*P* value
	*n*/*n* at risk	Incidence rate/10 000/year	95% CI	*n*/*n* at risk	Incidence rate/10 000/year	95% CI	Rate ratio	95% CI	
Any cancer	13/77	135.66	78.77–233.63	32/300	74.59	52.75–105.48	1.82	0.96–3.47	0.065
Breast cancer^a^	5/55	71.99	29.96–172.95	5/216	16.15	6.72–38.81	4.46	1.29–15.39	**0.010**
Kidney cancer	3/79	29.64	9.56–91.90	0/315	0.00	0.00–8.00	NA		**<0.001**
Lymphatic and haematopoietic cancers	2/79	19.74	4.94–78.93	9/315	19.76	10.28–37.97	1.00	0.22–4.62	0.999
Prostate cancer^b^	1/23	32.75	4.61–232.47	6/92	44.36	19.93–98.73	0.74	0.09–6.13	0.778
Colon cancer	0/78	0.00	0.00–36.06	4/312	8.74	3.28–23.28	0.00		0.346
Malignant melanoma	0/79	0.00	0.00–35.85	3/316	6.56	2.12–20.34	0.00		0.413
Gastric cancer	0/79	0.00	0.00–35.85	2/315	4.36	1.09–17.44	0.00		0.504
Lung and tracheal cancers	0/79	0.00	0.00–35.85	2/316	4.34	1.09–17.36	0.00		0.505
Ovarian cancer^a^	0/55	0.00	0.00–51.44	2/220	6.31	1.58–25.21	0.00		0.502
Skin cancer (other than melanoma)	0/79	0.00	0.00–35.85	2/316	4.35	1.09–17.38	0.00		0.505
Other cancers^c^	3/79	29.78	9.60–92.33	6/315	13.12	5.90–29.21	2.27	0.57–9.07	0.233

Bold values indicate a statistically significant difference between the patients and the controls (*P*  < 0.05, Mantel–Haenszel method). When the Bonferroni correction for multiple comparisons is applied, a *P* value < 0.004 (<0.05/12) is considered statistically significant.

^a^Only females included; ^b^Only males included; ^c^Other cancers in the patients included cancers of the thyroid gland, uterus, and pancreas (*n* = 1 each).

NA, not applicable.

### Long-term survival

**Figure 2:**
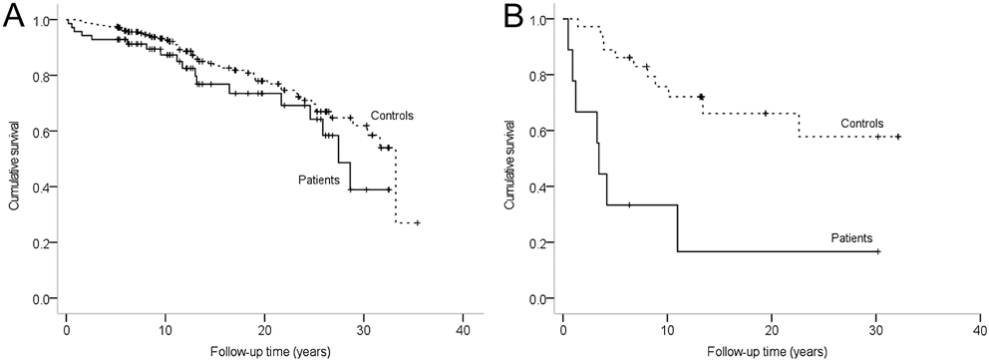
(A and B) Survival of the patients diagnosed with a non-metastatic (A) or a metastatic insulinoma (B) in Finland during 1980–2010, compared with controls matched for age, gender, and the place of residence (log-rank test). (A) Patients with non-metastatic insulinoma vs controls (*P*  = 0.125), (B) patients with metastatic insulinoma vs controls (*P*  < 0.001).

With the three disease progressions and three recurrences detected in the 71 patients treated with curative-intent surgery (6), the 5-, 10-, and 15-years disease-free survival rates were 94, 93, and 90%, respectively. During the follow-up, 25 (32%) patients and 63 (20%) controls deceased. The Kaplan–Meier survival curves of the patients with a non-metastatic or a metastatic insulinoma and their controls are shown in Fig. 2. In a Cox regression analysis, the median overall survival of 27.5 (95% CI: 24.1–30.8) years in the patients with a non-metastatic insulinoma did not significantly differ from the 33.2 (29.8–36.7) years in their controls (HR: 1.5 (0.9–2.6), *P*  = 0.128). In the patients with a metastatic insulinoma, the survival was significantly impaired, with a median of 3.4 (2.9–4.0) years vs not reached in the controls (HR: 5.1 (1.9–13.3), *P*  = 0.001). Three of the patients with metastatic insulinoma (33%), however, showed a remarkably long survival time of 6–30 years.

In univariate analyses, older age, distant metastases, tumour size ≥ 2 cm, higher preoperative serum insulin concentration, lack of curative-intent surgery, and the need for postoperative medication for the insulinoma were all associated with a significantly decreased overall survival among insulinoma patients (Supplementary Table 3). The occurrence of major surgical complications, classified as grades III–V of the Clavien–Dindo classification (8, 9), was associated with a decreased survival, due to the early postoperative mortality. After the exclusion of postoperative deaths (grade V complication), no significant difference was found in the survival of patients with major vs no or minor surgical complications (HR 2.28 (0.77–6.71), *P*  = 0.136). The association of laparoscopic vs open surgery with survival could not be assessed as only two patients underwent laparoscopic insulinoma surgery. In the multivariate analyses, older age and distant metastases were associated with decreased survival (Table 4 and Supplementary Table 4).

**Table 4:** Multivariate analysis of factors associated with mortality among patients diagnosed with an insulinoma in Finland during 1980–2010 (*n* = 75^a^).

Variable	Hazard ratio	95% CI	*P* value
Age at diagnosis	1.05	1.02–1.08	**0.003**
Tumour localization (head/neck vs body/tail)	1.90	0.72–5.03	0.197
Tumour size (≥2 cm vs <2 cm)	2.49	0.93–6.65	0.070
Distant metastases	3.71	1.18–11.67	**0.025**

Bold values indicate a statistically significant hazard ratio (*P*  < 0.05, Cox proportional hazards model).

^a^Four of the 79 patients in the total cohort were excluded from this multivariate analysis, due to missing data regarding tumour size (*n* = 4) and localization (*n* = 2).

### Causes of death

Nine of the 25 insulinoma patients who deceased during the study period (36%) died due to an insulinoma-related cause: 6 patients died of metastatic insulinoma, 2 patients died of surgical complications, and 1 patient died due to complications of invasive diagnostics, as previously described (6). With the two deaths due to surgical complications, the perioperative mortality, defined as any death occurring within 30 days after surgery, was 2.7%. The causes of death of the patients and controls are presented in Table 5. During the follow-up, 16 non-insulinoma-related deaths occurred in the patients and 54 in the controls. Of these 16 deaths among the patients, 8 (50%) were due to diseases of the circulatory system, 4 (25%) due to cancer and 4 (25%) due to other causes, compared to 24 (44%), 12 (22%) and 18 (33%) in the controls, respectively (*P*  = 0.765). The distribution of the causes of death did not significantly differ between the patients and the controls (*P*  = 0.363), analysed according to the national time-series classification of Statistics Finland.

**Table 5:** Causes of death of the patients diagnosed with an insulinoma in Finland during 1980–2010 and their control group matched for age, gender, and the place of residence. Data are presented as *n* (%).

	Patients (*n* = 79)	Controls (*n* = 316)
Deaths related to insulinoma	9 (11.4)	0 (0)
Deaths due to metastatic insulinoma	6 (7.6)	0 (0)
Deaths due to complications of the invasive diagnostics or pancreatic surgery	3 (3.8)	0 (0)
Deaths due to diseases of the circulatory system	8 (10.1)	29 (9.2)
Deaths due to tumours (other than insulinoma)	4 (5.1)	13 (4.1)
Deaths due to other causes	4 (5.1)	21 (6.6)
Alive at the end of follow-up	54 (68.4)	253 (80.1)

## Discussion

This study suggests an increased long-term morbidity in insulinoma patients, due to atrial fibrillation, intestinal obstruction, and possibly breast and kidney cancers. Endocrine and pancreatic diseases were more frequent within the first year after the diagnosis, likely due to a detection bias and the occurrence of short-term surgical complications, respectively. Despite the increased long-term morbidity, the overall survival of patients with non-metastatic insulinoma is similar to the general population. In patients with metastatic insulinoma, the prognosis is significantly impaired.

The long-term morbidity due to any cardiovascular disease, or due to atrial fibrillation was increased among the patients previously diagnosed with an insulinoma. The reason for the increased cardiovascular morbidity in insulinoma patients is unclear. Previous studies have found no association between insulinoma and hypertension (10, 11), but the incidence of atrial fibrillation in insulinoma patients has, to our knowledge, not been studied before. Hypoglycaemia has been shown to induce cardiac arrhythmias in persons with diabetes, but the potential effect of hypoglycaemia on the cardiovascular morbidity of insulinoma patients is unclear (12, 13). Unfortunately, the total burden of hyperinsulinaemia and hypoglycaemia could not be quantified retrospectively in the present study, nor in the previous ones. In our study, the follow-up due to insulinoma may have contributed to the early diagnoses of atrial fibrillation in the patients as the difference between the patients and the controls was no longer statistically significant after excluding the first year after diagnosis.

Morbidity due to any endocrine or thyroid disorders was increased among the patients during the first year after insulinoma diagnosis, but not later on. Although the diagnosis codes for hyperinsulinism and hypoglycaemia were excluded, the substantial number of other or unspecified endocrine disorders diagnosed near the time of diagnosis of insulinoma indicates that these codes may have been used instead of the specific diagnoses for insulinoma. A possible explanation for the increased thyroid morbidity is the careful examination and follow-up of these patients by endocrinologists, contributing to a prompt diagnosis of disorders that may partly remain undiagnosed in the general population.

The incidence of acute pancreatitis and intestinal obstruction was increased among the insulinoma patients vs controls. Most cases of pancreatitis occurred as early postoperative complications, with a rate of 10% in the surgically treated patients, as described previously (6). Intestinal obstruction occurred in 5% of the surgically treated patients, likely as a late postoperative complication of insulinoma surgery. Previous studies on surgically treated insulinomas have mainly focused on short-term complications and have not reported the incidence of late intestinal obstruction in insulinoma patients. In accordance with this study, previous studies have reported similar rates of intestinal obstruction after pancreatic and other abdominal surgery (14, 15, 16).

Morbidity due to breast and kidney cancers also seemed to increase among insulinoma patients. To our knowledge, increased incidence of breast or kidney cancers in insulinoma patients has not been reported before, apart from an increased risk for breast cancer in MEN1 patients (17). The breast and kidney cancers of the patients were diagnosed 2.7–24.3 years after the diagnosis of insulinoma. The MEN1 syndrome explained at least part of the increased morbidity due to breast cancer, and no statistically significant difference was found in the breast cancer incidence of the patients vs controls after the exclusion of MEN1 patients from the analysis. As renal cancers are often discovered incidentally in abdominal imaging (18, 19), we cannot exclude neither a true association of kidney cancer with insulinoma nor an effect of frequent CT scanning. As these results were based on a small number of patients diagnosed with breast or kidney cancer, larger studies are needed to confirm these preliminary findings.

No significant difference was detected in the incidence of dementia or other psychiatric disorders between insulinoma patients and controls. The sample size and the follow-up time in our study may, however, have been insufficient to detect a possible difference. A recent prospective study reported cognitive impairment in 18 of 34 insulinoma patients, measured with the Montreal Cognitive Assessment questionnaire prior to the pancreatic surgery, with improvement detected in most patients at 1 year after surgery (20).

In the present study, the overall survival of patients with non-metastatic insulinoma did not significantly differ from the general population. The 10-year survival of 87% for non-metastatic and 33% for metastatic insulinomas in this study was similar to the 91 and 29%, respectively, reported previously (1). Similarly to previous studies, older age and metastases were the most important factors associated with an impaired survival (1, 21, 22). In fact, recent evidence suggests that metastatic and non-metastatic insulinomas differ in their origin and pathogenesis and should be regarded as two different diseases (23). An increased risk of insulinoma recurrence and an impaired survival has been reported in MEN1 patients (1). In our study, however, no recurrences were detected during the follow-up of the two MEN1 patients.

Among the surgically treated insulinoma patients, the surgical method or the period of surgery was not associated with the overall survival. The postoperative mortality of 2.7% was slightly lower than the 3.7% reported previously for an open approach (4). Except for the postoperative deaths, no significant association was found between surgical complications and overall survival, which is in line with a recent series of 105 surgically managed pancreatic neuroendocrine tumour patients (24). A recent study of 198 insulinoma patients, however, reported a higher reoperation rate after tumour enucleations compared to pancreatic resections (25). To minimize the complication risks and need for reoperations, the invasive diagnostics and surgical treatment of insulinomas, like all pancreatic tumours, should be performed in centres with adequate expertise (2, 26, 27, 28, 29, 30, 31).

In this study, the median overall survival of 3.4 years in patients with a metastatic insulinoma was similar to the 40 months (3.3 years) reported recently in 31 patients with metastatic insulinomas (32). This is better than the median survival of 29 months (2.4 years) reported earlier (22). Despite the poor overall survival, one-third of the patients with metastatic insulinoma had a remarkably long survival time. Previous studies have shown that palliative debulking surgery, and newer treatment options, such as peptide receptor radionuclide therapy and everolimus, may improve survival and relieve symptoms in patients with metastatic disease (26, 32, 33, 34, 35, 36). Because of the small number of metastatic insulinomas, we were not able to assess the effect of treatment on the survival of patients with metastatic insulinoma.

The major strengths of this study are the nationwide, unselected study cohort, including all the patients diagnosed with an insulinoma in Finland over a 3-decade period, and the long-term follow-up data of the patients and controls, matched for age, gender, and the place of residence. In Finland, it is mandatory to report the underlying causes of death to the Population Information System, and the hospital discharge diagnoses to the National Hospital Discharge Register, contributing to the complete, comprehensive, and high-quality data in these registers (37, 38).

The relatively small sample size, due to the rarity of insulinomas, is the major limitation of this study. In addition, the prognosis of the subgroups of patients with metastatic, recurrent, or MEN1-related insulinomas could not be evaluated comprehensively. Due to the retrospective, register-based study design we were not able to specify the causative factors of the long-term morbidity in insulinoma patients. Another limitation is that the National Hospital Discharge Register only includes information on the hospital visits in the specialized health care system, which may lead to underestimation of the incidence of non-severe diseases, treated mainly in the primary health care. On the other hand, detection bias probably contributed to the high incidence of non-severe endocrine disorders near the time of diagnosis of insulinoma in the patients.

In conclusion, the long-term prognosis of patients with a non-metastatic insulinoma seems to be similar to the general population, except for an increased incidence of atrial fibrillation, intestinal obstruction, and possibly breast and kidney cancers. Metastatic insulinomas are rare but generally entail a markedly decreased survival. To our knowledge, this is the first study to report findings of increased long-term morbidity in insulinoma patients. In the future, larger studies are needed to confirm these results.

## Supplementary Material

Supplementary Table 1. Classification of endocrine, cardiovascular, gastrointestinal, and mental and behavioural disorders, according to the Finnish version of the ICD-10 since 1996, ICD-9 during 1987–1995, and ICD-8 during 1980–1986.

Supplementary Table 2. Prevalence of endocrine, cardiovascular, gastrointestinal, psychiatric disorders and cancers in the 79 insulinoma patients diagnosed with an insulinoma in Finland during 1980–2010, and in 316 matched controls, before the diagnosis of insulinoma.

Supplementary Table 3. Univariate Cox regression analysis of factors associated with mortality in the 79 patients diagnosed with an insulinoma in Finland during 1980–2010.

Supplementary Table 4. Multivariate analysis of factors associated with mortality in surgically treated insulinoma patients (n=73).

## Declaration of interest

The authors declare that there is no conflict of interest that could be perceived as prejudicing the impartiality of this study.

## Funding

This work was supported by the Competitive State Research Financing of the Expert Responsibility Area of Tampere University Hospital (grant numbers 9U012, 5900/3225, 6000/3231; to E P, P J), the Medical Research Fund of Seinäjoki Central Hospital (grant numbers 1717/6043, 1717/6080; to E P, P J), the Seinäjoki City and Tampere University Research Funds (not numbered; to E P, P J), the Helsinki University Hospital Research Funds (grant number TYH2019254; to C S-J) and Finska Läkaresällskapet (not numbered; to C S-J). The funders had no role in study design, data collection or analysis, decision to publish, or preparation of the manuscript. Preliminary results of this study have been presented as an e-poster at the eECE2021 congress (39).
