# Supplementary material for: Long-term morbidity and mortality in patients diagnosed with an insulinoma
Source: Eur J Endocrinol. 2021 Sep 1;185(4):577–86. doi: 10.1530/EJE-21-0230 (PMC8784472; doi:10.1530/EJE-21-0230)
Supplement: Supplementary Table 1. Classification of endocrine, cardiovascular, gastrointestinal, and mental and behavioural disorders, according to the Finnish version of the ICD-10 since 1996, ICD-9 during 1987–1995, and ICD-8 during 1980–1986. [file supplementary_table_1.pdf]

Supplementary Table 1. Classification of endocrine, cardiovascular, gastrointestinal, and mental and behavioural disorders, according to the Finnish version of the ICD-10 since 1996, ICD-9 during 1987–1995, and ICD-8 during 1980–1986.

	Classification of diseases		
Disease category	ICD-8	ICD-9	ICD-10
Endocrine disorders[a]	240–250, 252–258	240–250, 252–259	E00–E14, E20–E35
Diabetes	250	250	E10–E14
Thyroid disorders	240–246	240–246	E00–E07
Parathyroid disorders	252	252	E20–E21
Other endocrine disorders[b]	253–258	253–258	E22–E29, E31–E35
Cardiovascular diseases[c]	400–414, 423–458	401–417, 423–459	I10–I28, I31, I34–I37, I42–I99
Cerebrovascular diseases	430–438	430–438	I60–I69
Hypertension	400–404	401–405	I10–I15
Arrhythmias and conduction disorders	427,2–427,98	426–427	I44–I49
Atrial fibrillation and flutter	427,92	4273A	I48
Coronary artery disease	410–414	410–414	I20–I25
Diseases of the arteries and veins	440–448, 451–458	440–448, 451–459	I70–I89
Valvular diseases and cardiomyopathies	423–425	423–425	I31, I34–I37, I42–I43
Heart failure	427.0, 427.1, 428	428	I50
Diseases of the pulmonary circulation	426, 450	415–417	I26–I28
Gastrointestinal diseases	530–577	530–579	K20–K93
Diseases of the oesophagus, stomach, and duodenum	530–537	530–537	K20-K31
Abdominal hernias	550–553	550–553	K40–K46
Chronic inflammatory bowel diseases	563	555–556	K50–K51
Diseases of the appendix	540–543	540–543	K35–K38
Other bowel diseases[d]	560–562, 564–569	557–569	K52–K67, K90–K93
Diseases of the liver, biliary tract, and gallbladder	570–576	570–576	K70–K83
Diseases of the pancreas	577	577	K85–K87
Mental and behavioural disorders	290–315	290–315	F00–F99
Dementia	290	290	F00–F03

[a]Excluding hyperinsulinism and hypoglycemia, [b]Including pituitary, thymic, adrenal, ovarian, testicular, polyglandular and other or unspecified endocrine disorders, [c]Excluding rheumatic heart diseases and infectious endo-, peri- and myocardial diseases.

ICD International Classification of Diseases
