# Supplementary material for: Long-term morbidity and mortality in patients diagnosed with an insulinoma
Source: Eur J Endocrinol. 2021 Sep 1;185(4):577–86. doi: 10.1530/EJE-21-0230 (PMC8784472; doi:10.1530/EJE-21-0230)
Supplement: Supplementary Table 2. Prevalence of endocrine, cardiovascular, gastrointestinal, psychiatric disorders and cancers in the 79 insulinoma patients diagnosed with an insulinoma in Finland during 1980–2010, and in 316 matched controls, before the diagnosis of insulinoma. [file supplementary_table_2.pdf]

Supplementary Table 2. Prevalence of endocrine, cardiovascular, gastrointestinal, psychiatric disorders and cancers in the 79 insulinoma patients diagnosed with an insulinoma in Finland during 1980–2010, and in 316 matched controls, before the diagnosis of insulinoma.

	Patients (n = 79)		Controls (n = 316)		Patients vs controls		
	n	(%)	n	(%)	Odds Ratio	(95% CI)	Significance
Endocrine disorders[a]	7	8.9	15	4.7	1.94	0.76–4.91	0.163
Diabetes	4	5.1	6	1.9	2.67	0.75–9.45	0.129
Thyroid disorders	0	0.0	5	1.6	0.00	NA	NA
Parathyroid disorders	0	0.0	1	0.3	0.00	NA	NA
Other endocrine disorders[b]	3	3.8	3	0.9	4.00	0.81–19.82	0.090
Cardiovascular diseases	20	25.3	62	19.6	1.45	0.78–2.69	0.237
Cerebrovascular diseases	1	1.3	7	2.2	0.57	0.07–4.64	0.601
Hypertension	9	11.4	18	5.7	2.18	0.92–5.13	0.075
Arrhythmias and conduction disorders	2	2.5	9	2.8	0.89	0.19–4.11	0.880
Atrial fibrillation and flutter	1	1.3	5	1.6	0.80	0.09–6.85	0.839
Coronary artery disease	2	2.5	12	3.8	0.65	0.14–3.02	0.582
Disease of the arteries and veins	6	7.6	35	11.1	0.65	0.26–1.63	0.362
Valvular diseases and cardiomyopathies	3	3.8	3	0.9	7.81	0.77–78.82	0.082
Heart failure	2	2.5	4	1.3	2.17	0.35–13.47	0.404
Diseases of the pulmonary circulation	0	0.0	0	0.0	NA	NA	NA
Gastrointestinal diseases	12	15.2	46	14.6	1.05	0.52–2.14	0.884
Diseases of the oesophagus, stomach, and duodenum	3	3.8	6	1.9	2.00	0.50–8.00	0.327
Abdominal hernias	6	7.6	13	4.1	2.03	0.70–5.84	0.190
Chronic inflammatory bowel diseases	0	0.0	1	0.3	0.00	NA	NA
Diseases of the appendix	1	1.3	8	2.5	0.50	0.06–4.00	0.513
Other bowel diseases	2	2.5	12	3.8	0.66	0.14–3.00	0.589
Diseases of the liver, biliary tract, and gallbladder	2	2.5	16	5.1	0.48	0.11–2.16	0.342
Diseases of the pancreas	1	1.3	1	0.3	4.00	0.25–63.95	0.327
Mental and behavioural disorders	8	10.1	22	7.0	1.53	0.64–3.64	0.339
Dementia	0	0.0	4	1.3	0.00	NA	NA
Cancers	2	2.5	8	2.5	1.00	0.20–4.90	1.000

[a]Excluding hyperinsulinism and hypoglycemia, [b]Other endocrine disorders in the patient group included a pituitary disorder (n=1), and other or unspecified endocrine disorders (n=2).

CI Confidence Interval. NA not applicable. Bold text indicates a statistically significant Odds ratio (p<0.05, Conditional logistic regression).
