# Supplementary material for: Long-term morbidity and mortality in patients diagnosed with an insulinoma
Source: Eur J Endocrinol. 2021 Sep 1;185(4):577–86. doi: 10.1530/EJE-21-0230 (PMC8784472; doi:10.1530/EJE-21-0230)
Supplement: Supplementary Table 3. Univariate Cox regression analysis of factors associated with mortality in the 79 patients diagnosed with an insulinoma in Finland during 1980–2010. [file supplementary_table_3.pdf]

Supplementary Table 3. Univariate Cox regression analysis of factors associated with mortality in the 79 patients diagnosed with an insulinoma in Finland during 1980–2010.

	Hazard Ratio	95% CI	Significance
Clinical factors			
Age at diagnosis (n = 79)	1.05	1.02–1.08	**0.001**
Male (n = 24) vs. female (n = 55)	1.03	0.44–2.39	0.955
Symptom duration before diagnosis (n = 75)	1.00	1.00–1.01	0.293
Decade of diagnosis (reference: 2000s, n = 43)			
1990s (n = 17)	1.58	0.55–4.59	0.397
1980s (n = 19)	1.17	0.37–3.67	0.793
Preoperative laboratory tests			
Lowest spontaneous plasma glucose, mmol/l (n = 62)	2.03	0.85–4.82	0.111
Highest spontaneous serum insulin, mU/l (n = 49)[a]	1.10	1.01–1.20	**0.037**
Highest spontaneous serum C-peptide, nmol/l (n = 39)	1.74	0.99–3.04	0.053
Plasma glucose nadir in the fasting test, mmol/l (n = 64)	1.10	0.58–2.11	0.770
Corresponding serum insulin in the fasting test, mU/l (n = 55)	1.01	0.99–1.03	0.265
Corresponding serum C-peptide in the fasting test, nmol/l (n = 44)	1.04	0.40–2.72	0.939
Glycated haemoglobin, HbA1c, % (n = 31)	0.97	0.23–4.12	0.971
Treatment-related factors			
Surgery (reference: curative-intent surgery, n = 71)			
Palliative surgery (n = 2)	17.01	3.45–83.92	**0.001**
No surgery (n = 6)	9.86	3.60–27.00	**<0.001**
Surgical method (reference: enucleation, n = 31)			
Distal resection (n = 33)	1.21	0.44–3.30	0.717
Pancreatico-duodenectomy (n = 9)	2.11	0.54–8.23	0.283
Major surgical complications[b]: yes (n = 18) vs. no (n = 55)	3.06	1.15–8.12	**0.025**
Preoperative medication[c]: yes (n = 55) vs. no (n = 24)	0.97	0.42–2.24	0.934
Postoperative medication[d] yes (n = 19) vs. no (n = 53)	3.09	1.23–7.75	**0.016**
Postoperative diazoxide: yes (n = 8) vs. no (n = 63)	4.56	1.59–13.03	**0.005**
Postoperative somatostatin analogue: yes (n = 19) vs. no (n = 54)	2.04	0.84–4.95	0.113
Tumour-related factors			
Tumour size ≥ 2 cm (n = 20) vs. < 2 cm (n = 55)	2.79	1.19–6.55	**0.018**
Multiple (n = 5) vs. solitary tumour (n = 73)	0.67	0.09–5.05	0.700
Tumour localization: head/neck (n = 39) vs. body/tail (n = 38)	0.97	0.43–2.21	0.949
Distant metastases: yes (n = 9) vs. no (n = 70)	5.06	2.06–12.41	**<0.001**
Clinical TNM staging (7) (reference: Stage I, n = 58)			
II (n = 10)	1.88	0.53–6.66	0.328
III (n = 0)			
IV (n = 9)	5.06	1.99–12.88	**0.001**

[a]Hazard Ratio calculated for a 10mU/l rise in plasma insulin concentration, [b]Classified as grades III–V of the Clavien-Dindo classification (8, 9), [c]Including medical treatment of inoperable patients,

[d]Including diazoxide (n=8), somatostatin analogue (n=19), peroral corticosteroids (n=3), interferon alpha (n=3), 5-fluorouracil-streptozotocin (n=2), streptozotocin (n=1), epirubicin (n=1), doxorubicin (n=1), and sunitinib (n=1).

CI Confidence Interval. Bold text indicates a statistically significant hazard ratio (p<0.05, Cox proportional hazards model).
