# Supplementary material for: Long-term morbidity and mortality in patients diagnosed with an insulinoma
Source: Eur J Endocrinol. 2021 Sep 1;185(4):577–86. doi: 10.1530/EJE-21-0230 (PMC8784472; doi:10.1530/EJE-21-0230)
Supplement: Supplementary Table 4. Multivariate analysis of factors associated with mortality in surgically treated insulinoma patients (n=73). [file supplementary_table_4.pdf]

Supplementary Table 4. Multivariate analysis of factors associated with mortality in surgically treated insulinoma patients (n=73).

Variable	Hazard Ratio	95% CI	Significance
Age at surgery	1.08	1.03–1.13	**0.001**
Surgical method (reference: enucleation)			
Distal resection	0.81	0.27–2.44	0.704
Pancreatico-duodenectomy	4.07	0.80–20.87	0.092
Time period of surgery (reference: 1980–1989)			
1990–1999	1.20	0.35–4.12	0.775
2000–2010	0.39	0.09–1.73	0.213
Distant metastases	4.58	1.29–16.25	**0.018**

CI Confidence Interval. Bold text indicates a statistically significant hazard ratio ($p<0.05$, Cox proportional hazards model
